# Telomere- and Telomerase-Associated Proteins and Their Functions in the Plant Cell

**DOI:** 10.3389/fpls.2016.00851

**Published:** 2016-06-28

**Authors:** Petra Procházková Schrumpfová, Šárka Schořová, Jiří Fajkus

**Affiliations:** ^1^Mendel Centre for Plant Genomics and Proteomics, Central European Institute of Technology, Masaryk UniversityBrno, Czech Republic; ^2^Laboratory of Functional Genomics and Proteomics, National Centre for Biomolecular Research, Faculty of Science, Masaryk UniversityBrno, Czech Republic; ^3^Institute of Biophysics, Academy of Sciences of the Czech Republic, v.v.i.Brno, Czech Republic

**Keywords:** telomere, telomerase, telomeric proteins, shelterin, telomeric repeat binding (TRB), plant

## Abstract

Telomeres, as physical ends of linear chromosomes, are targets of a number of specific proteins, including primarily telomerase reverse transcriptase. Access of proteins to the telomere may be affected by a number of diverse factors, e.g., protein interaction partners, local DNA or chromatin structures, subcellular localization/trafficking, or simply protein modification. Knowledge of composition of the functional nucleoprotein complex of plant telomeres is only fragmentary. Moreover, the plant telomeric repeat binding proteins that were characterized recently appear to also be involved in non-telomeric processes, e.g., ribosome biogenesis. This interesting finding was not totally unexpected since non-telomeric functions of yeast or animal telomeric proteins, as well as of telomerase subunits, have been reported for almost a decade. Here we summarize known facts about the architecture of plant telomeres and compare them with the well-described composition of telomeres in other organisms.

## Telomeres As Nucleoprotein Structures

Telomeres are nucleoprotein structures at the ends of eukaryotic chromosomes that protect linear chromosomes against damage by endogenous nucleases and erroneous recognition as unrepaired chromosomal breaks. It is now known that telomeric structures are formed by telomeric DNA, histone octamers, and a number of proteins that bind telomeric DNA, either directly or indirectly, and together, form the protein telomere cap (Fajkus and Trifonov, 2001; de Lange, 2005; Bianchi and Shore, 2008; Sfeir, 2012). The telomeric cap proteins of diverse organisms are less conserved than one might expect. Even within a single taxonomic class, such as mammals, telomeric proteins display less conservation than other chromosomal proteins (Linger and Price, 2009). On the other hand, in many plant families, whole-genome duplication events have occurred, resulting in a multitude of genomic changes, such as deletions of large fragments of chromosomes, silencing of duplicate genes, and recombining of homologous chromosomal segments, as was shown, e.g., in crucifer species (Mandakova and Lysak, 2008). Polyploidy can result in increased numbers of genes of the same family (Taylor and Raes, 2004; He and Zhang, 2005; Freeling, 2009), which may show sub-functionalization, neo-functionalization, and partial or full redundancy and complicates assignment of an actual and specific function for individual proteins *in vivo*. Gene duplications and losses in plant phylogeny can be traced also in telomere associated protein families (e.g., in *Arabidopsis thaliana*: single myb histone (SMH) family, TRF-like (TRFL) family, or Pot1-like family) (Nelson et al., 2014; Beilstein et al., 2015).

**FIGURE 1:**
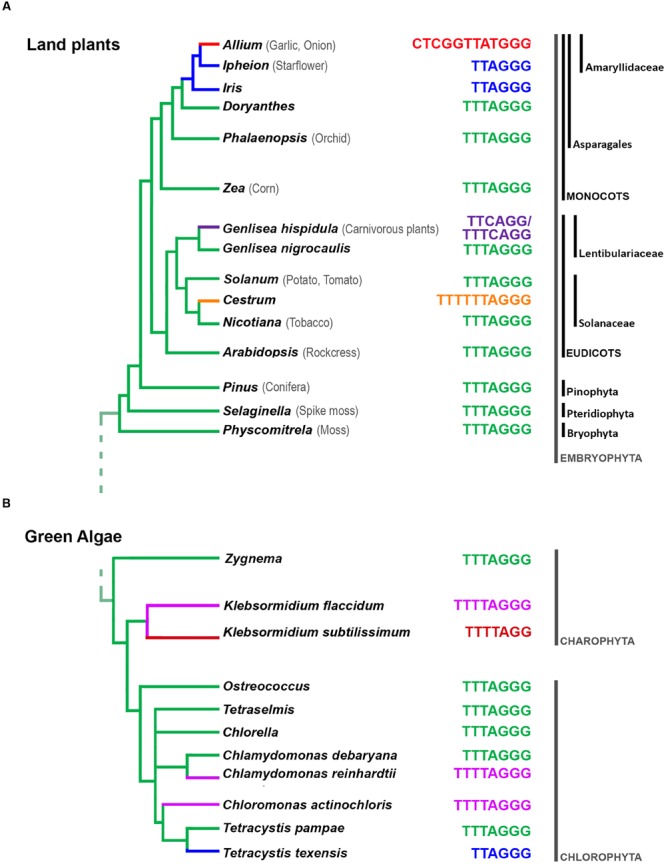
**Summary of current knowledge on telomere DNA diversity in land plants (A) and green algae (B).** The prevalent plant telomeric sequence motif TTTAGGG was first described in *A. thaliana* (Richards and Ausubel, 1988). Divergent telomeric sequences have been observed in Asparagales (Sykorova et al., 2003), in *Cestrum* spp. (Peska et al., 2015), in *Genlisea* (Tran et al., 2015), or in *Allium* (Fajkus et al., 2016). While *Arabidopsis*-type telomeric sequence is dominant in “green lineages” of algae Chlorophyta and Streptophyta, this ancestral motif was replaced several times with a novel motifs (reviewed in Fulnečková et al., 2015). (The relationships between families and genera are adapted from the schematic phylogenetic tree presented in Fulnečková et al., 2015 and Fajkus et al., 2016.)

In land plants, the telomere is mostly composed of *Arabidopsis*-type TTTAGGG repeats (Richards and Ausubel, 1988; **Figure 1A**). Known exceptions are species in the order Asparagales, starting from divergence of the Iridaceae family, which shares the human-type telomeric repeat (TTAGGG; probably caused by a mutation that altered the RNA template subunit of telomerase ∼80 Mya; Adams et al., 2001; Weiss and Scherthan, 2002; Sykorova et al., 2003). The human-type telomere is also shared by species of the Allioideae subfamily, except for the *Allium* genus (Sykorova et al., 2006), where novel telomeric sequence (CTCGGTTATGGG) was recently described (Fajkus et al., 2016). An unusual telomeric motif (TTTTTTAGGG) was found in the family Solanaceae, in *Cestrum elegans* and related species (Peska et al., 2015). Also some of the species from the carnivorous genus *Genlisea* display, instead normal *Arabidopsis*-type of telomere, two intermingled sequence variants (TTCAGG and TTTCAGG; Tran et al., 2015).

Moreover, across the Plantae kingdom, outside of land plants but including red algae, green algae, and Glaucophytes (Koonin, 2010), telomere types also vary (**Figure 1B**). For example, in algae, in addition to the *Arabidopsis*-type of telomeric repeat, the *Chlamydomonas*-type (TTTTAGGG), human-type (TTAGGG), and a novel TTTTAGG repeat have been described (Fulnecková et al., 2013; Fulnečková et al., 2015).

The length of plant telomeric DNA at a single chromosomal arm can be as small as 500 base pairs (bp) in *Physcomitrella patens* (Shakirov et al., 2010; Fojtova et al., 2015), as long as 160 kb in *Nicotiana tabacum* (Fajkus et al., 1995), or 200 kb in *Nicotiana sylvestris* (Kovarik et al., 1996). Besides the remarkable variation in telomere lengths among diverse plant genera or orders, telomere lengths can also vary at the level of the species or ecotypes: e.g., *Arabidopsis* telomeres range from 1.5 to 9 kb, depending on the ecotype. Also in the long-living organism *Betula pendula*, telomeres in different genotypes varied from a minimum length of 5.9–9.6 kb to a maximum length of 15.3–22.8 kb (Shakirov and Shippen, 2004; Maillet et al., 2006; Aronen and Ryynanen, 2014).

Since telomeric DNA serves as a landing pad for a set of proteins, the total length or composition of telomeric tracts could markedly affect the number or selection of telomere-associated proteins and subsequently influence telomere packaging, structural transitions, or launch various biochemical pathways (see below).

## Nuclear Localization and Dynamics of Telomeres

In some species during interphase, telomeres, and centromeres could be located at opposite sides of the nucleus, at the nuclear periphery, in limited regions or clusters; this is known as the Rabl organization (Rabl, 1885; for review, see Cowan et al., 2001). The Rabl organization (Wen et al., 2012) was observed in wheat, rye, barley, and oats. Other plant species, such as maize (*Zea mays*) and sorghum (*Sorghum bicolor*), despite having fairly large genomes, are not known to exhibit the Rabl configuration (Dong and Jiang, 1998). A recent study among *Brachypodium* species revealed a positive correlation between Rabl configuration and an increase in DNA content (resulting from replication) and a negative influence of increasing nuclear elongation (Idziak et al., 2015). A rosette-like organization of chromosomes in interphase nuclei was observed in *Arabidopsis*: telomeres show persistent clustering at the nucleolus while centromeres do not cluster (Armstrong et al., 2001; Tiang et al., 2012). Moreover, during early meiotic prophase, at the leptotene–zygotene transition, telomeres of most plant species cluster to form a bouquet (Bass et al., 1997; Martinez-Perez et al., 1999; Cowan et al., 2002; Corredor and Naranjo, 2007; Higgins et al., 2012; Phillips et al., 2012). *Arabidopsis* belongs to a small group of species that do not form telomeric bouquets (Armstrong et al., 2001).

Chromatin attachment to the inner nuclear membrane in plants, as well as in other species, is mediated by a well conserved multi-protein complex gathered around SUN (Sad1-UNC-84 homology)-KASH (Klarsicht, ANC-1, and Syne homology) proteins [respectively AtSUN-AtSINE (SUN domain-interacting NE proteins) in *A. thaliana*; Starr et al., 2001; Zhou et al., 2014; Tamura et al., 2015]. In fission and budding yeasts, interactions during meiosis between telomeres and the nuclear envelope, via interactions between SUN domain proteins and telomere-binding proteins, was described: in *Saccharomyces cerevisiae* SUN-domain protein yMps3 (monopolar spindle protein 3) is needed for yKu80-mediated telomeric chromatin anchoring (Schober et al., 2009), while in *Schizosaccharomyces pombe*, interactions between telomeric protein pRap1 (repressor activator protein 1) and pSUN proteins are mediated by pBqt1 and pBqt2 (telomere bouquet protein 1 and 2; Chikashige et al., 2006). The tethering of human telomeres to the nuclear matrix was proposed to depend on an isoform of telomere repeat binding factor 1 (TRF1) interacting partner (hTIN2), named hTIN2L (Kaminker et al., 2009), or an A-type lamin (Ottaviani et al., 2008; for review, see Giraud-Panis et al., 2013). Various homologs of SUN domain proteins were identified in *Arabidopsis* or in maize. In *Arabidopsis*, they are also localized to the inner nuclear membrane in somatic cells (Graumann et al., 2010; Tamura et al., 2015), however, homologs of Bqt proteins or TIN2 proteins have not been found in plants and their sequences are poorly conserved.

Telomeres are processed by a telomere-specific machinery that includes telomerase and its regulatory units, as well as nucleases, as exemplified by the exonuclease 1 (AtEXO1) ortholog in *Arabidopsis* (Kazda et al., 2012; Derboven et al., 2014). In plants, as well as in most of other kingdoms, replication of chromosomal ends results in single-stranded 3′ DNA protrusions (G-overhangs) after degradation of the last RNA primer at the 5′ terminus of a nascent strand. In *Silene latifolia* or *A. thaliana*, relatively short (20–30 nucleotides) G-overhangs were detected. Moreover, half of the Silene and *Arabidopsis* telomeres showed no overhangs or overhangs less than 12 nucleotides in length (Riha et al., 2000; Kazda et al., 2012). These G-overhangs are also thought to be required for chromosome end protection by forming secondary DNA structures such as t-loops (reviewed in Tomaska et al., 2009). Although formation of t-loop structures was demonstrated among plants only in the garden pea (*Pisum sativum*; Cesare et al., 2003), it is believed that excision from a t-loop in *Arabidopsis* may result in t-circle formation and in telomere rapid deletion (Watson and Shippen, 2007). In tobacco cell culture, knockdown of one of three human hnRNP homologs, named NgGTBP1 (G-strand specific single-stranded telomere-binding protein 1), led to frequent formation of extrachromosomal t-circles, inhibition of single-stranded invasion into double-stranded telomeric DNA and the loss of protection of telomeres against inter-telomeric recombination (Lee and Kim, 2010, 2013).

As well as in humans, mouse, or *Caenorhabditis* (Uringa et al., 2011; Vannier et al., 2012), the regulator of telomere elongation helicase 1 (AtRTEL1) plays a putative role in *Arabidopsis* in the destabilization of DNA loop structures such as t-loops or d-loops (Recker et al., 2014). However, a substantial portion of telomeres in *Arabidopsis* does not apparently undergo nucleolytic resection, and 3′ ends produced by leading-strand replication remain blunt-ended (Riha et al., 2000). It is believed that blunt-ends in *Arabidopsis* are specifically recognized and protected by the AtKu70/80 heterodimer although *in situ* localization of Ku to telomeres remains elusive (Kazda et al., 2012).

## Proteins Associated with Telomeric DNA

**FIGURE 2:**
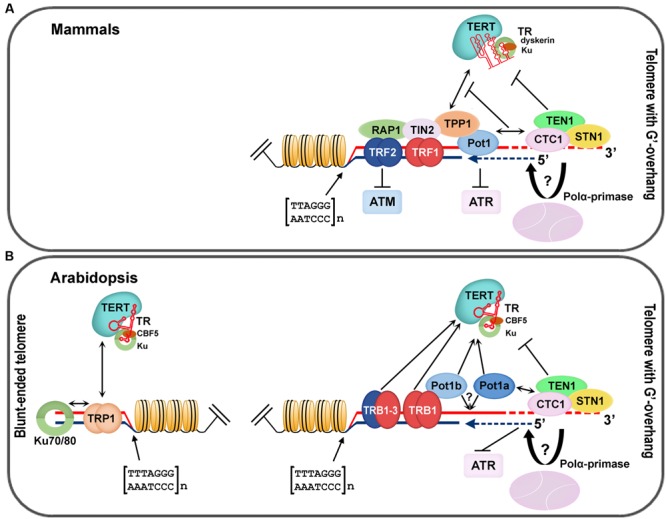
**Nucleoprotein complexes associated with mammalian and *A. thaliana* telomeres. (A)** Mammalian shelterin proteins (TRF1/2, Rap1, TIN2, TPP, and Pot1) modulate access to the telomerase complex and the ATR/ATM dependent DNA damage response pathway. The CST complex (CTC1–STN1–TEN1) affects telomerase and DNA polymerase α recruitment to the chromosomal termini, and thus coordinates G-overhang extension by telomerase with fill-in synthesis of the complementary C-strand (blue dashed line; figure adopted from Chen and Lingner, 2013). **(B)** *Arabidopsis* TRB1, 2 and 3 interact with the telomeric sequence due to the same Myb-like binding domain as mammalian TRF1/2 (Marian et al., 2003; Kuchar and Fajkus, 2004; Schrumpfova et al., 2004). TRB proteins interact with TERT and Pot1b and when localized at chromosomal ends they are eligible to function as components of the plant shelterin complex, mainly at telomeres with a G-overhang (Schrumpfova et al., 2008, 2014). An evolutionary conserved CST complex is suggested to coordinate the unique requirements for efficient replication of telomeric DNA in plants as well as in other organisms (Derboven et al., 2014). Blunt-ended telomeres are specifically recognized and protected by the KU70/80 heterodimer that directly interacts with TRP1, and by extension, with TERT (Kuchar and Fajkus, 2004; Kazda et al., 2012; Schrumpfova et al., 2014).

Telomere-associated proteins can regulate lengths of telomere tracts by modulating access of telomerase or affecting conventional DNA replication machinery. In mammals, telomeric DNA associates with a six-protein complex called shelterin. The specific telomeric dsDNA binding is mediated by TRF1 and TRF2 (Broccoli et al., 1997; Court et al., 2005), through their Myb-like domain with an LKDKWRT amino acid motif that is also conserved in other telobox binding proteins, not only in mammals but also in plants (Bilaud et al., 1996; Feldbrugge et al., 1997). A bridge between proteins directly associated with DNA—TRF1, TRF2, and ssDNA binding protein Pot1 (Protection of telomeres 1)—is mediated by TIN2 and the oligosaccharide/oligonucleotide binding (OB)-fold domain of TPP1 (TINT1, PTOP, PIP1) protein (for review see Schmidt and Cech, 2015; Lazzerini-Denchi and Sfeir, 2016). Moreover, protein Rap1, the last component of shelterin, interacts with TRF2 (Arat and Griffith, 2012) and modulates its recruitment to telomeric DNA (Janouskova et al., 2015). A schematic model of mammalian telomere-associated proteins (**Figure 2A**) and a proposed model of the telomeric complex in *A. thaliana* (**Figure 2B**) summarizes recent knowledge in mammalian and plant telomere biology and provides a clear comparison of conserved structures at chromosome termini. In addition, a general overview of telomere-associated proteins that have been described in plants is given in **Table 1**. Detailed description of telomeric and putative telomeric dsDNA and ssDNA binding proteins from *A. thaliana* is shown in **Table 2**.

**Table 1:** A general overview of telomere/telomerase associated proteins described in plants.

		*A. thaliana*	*O. sativa*	*Z. mays*	*N. glutinosa/N. tabacum/N. sylvestris*	*H. vulgare*	*S. lycopersicum/S. tuberosum*	*C. parqui*	*P. crispum*
**Telomeric dsDNA-associated proteins**							
Myb-like proteins	Myb-like domain at the N-terminus (SMH family)	AtTRB1-3 (Schrumpfova et al., 2004)	OsTRBF1-3 (Byun et al., 2008; He et al., 2013)	ZmSMHs (Marian et al., 2003)					PcMYB1 (Feldbrugge et al., 1997)
	
	Myb-like domain at the C-terminus (TRFL-family)	AtTBP1 (Hwang et al., 2001) AtTRP1 (Chen et al., 2001) AtTRFL2-10 (Karamysheva et al., 2004)	OsRTBP1 (Yu et al., 2000)	ZmIBP1 (Lugert and Werr, 1994) ZmIBP2 (Moore, 2009)	NgTRF1 (Yang et al., 2003)		LeTBP1 (Moriguchi et al., 2006)	CpTBP1 (Peska et al., 2011)	PcBPF-1 (da Costa e Silva et al., 1993)
	
	Myb-like domain at the C-terminus (AID family)		OsAID1 (Zhu et al., 2004; He et al., 2013)	ZmTacs1 (Marian and Bass, 2005)					

CST complex		AtStn1 (Song et al., 2008) AtTEN1 (Leehy et al., 2013) AtCTC1 (Surovtseva et al., 2009)							

**Telomeric ssDNA-associated proteins**							
OB-fold	Pot-like	AtPot1a-c (Kuchar and Fajkus, 2004; Tani and Murata, 2005; Rossignol et al., 2007)		ZmPot1a (Shakirov et al., 2009b) ZmPot1b (Shakirov et al., 2009b)		HvPot1 (Shakirov et al., 2009b)	StPot1 (Shakirov et al., 2009b)	CpPot1 (Peska et al., 2008)	

Non-OB fold	WHY	AtWhy1 (Yoo et al., 2007)				HvWhy1 (Grabowski et al., 2008)			
	
	RRM-motif	AtSTEP1 (Kwon and Chung, 2004)	Os08g0492100 (He et al., 2013) Os08g0320100 (He et al., 2013)		NtGTBP1-3 (Hirata et al., 2004; Lee and Kim, 2010)				

**Telomerase associated**							

TERT subunit		AtTERT (Fajkus et al., 1996)	OsTERT (Oguchi et al., 2004)	ZmTERT (Sykorova et al., 2009)	NtTERT, NsTERT (Sykorova et al., 2012)				

TERT/TR associated proteins	Myb-like domain at the N-terminus (SMH family)	AtTRB1-3 (Schrumpfova et al., 2014)							
	
	Myb-like domain at the C-terminus (TRFL family)	AtTRP1 (Schrumpfova et al., 2014)							
	
	Dyskerin-like	AtCBF5 (Lermontova et al., 2007)							
	
	Pot-like	AtPot1a (Rossignol et al., 2007)							
	
	RRM-motif	AtRRM (Lee L.Y. et al., 2012)							
	
	ARM-motif	AtARM (Lee L.Y. et al., 2012)							
	
	RNA-binding	AtG2p (Dokládal et al., 2015)							
	
	Metallothionein-like	AtMT2A (Dokládal et al., 2015)							

**DNA processing and repair-associated proteins at telomerases**							

Helicase		AtRTEL1 (Recker et al., 2014)							

Exonuclease		EXO1 (Kazda et al., 2012)							

Ku		AtKu70/80 (Bundock et al., 2002; Riha et al., 2002)	OsKu70 (Hong et al., 2010)						

PI3 kinase		AtATM (Amiard et al., 2011) AtATR (Amiard et al., 2011)							

MRN Complex		AtRad50 (Gallego and White, 2001) AtMre11 (Bundock and Hooykaas, 2002) AtNbs1 (Najdekrova and Siroky, 2012)							

Ku-independent EJ pathway		AtRad1 (Vannier et al., 2009) AtERCC1 (Vannier et al., 2009) AtXRCC1 (Amiard et al., 2014)							

Backup-NHEJ KU-independent pathway		AtPARP1, 2 (Boltz et al., 2014)							

*Proteins that were proven as telomeric DNA- or telomerase-associated proteins are listed above. Their homologues involved in non-telomeric processes, while their associations with telomeres or telomerase have not been observed, are shown in green. Single Myb Histone family (SMH); TRF-like (TRFL-family); Anther Indehiscence family (AID-family); Cdc13/CTC1-Stn1-Ten1 (CST); Oligonucleotide/Oligosaccharide Binding fold (OB-fold); proteins without Oligonucleotide/Oligosaccharide Binding fold (non-OB-fold); Protection of telomeres – like (Pot-like); Whirly (WHY); RNA Recognition Motifs (RRM-motif); Telomerase Reverse Transcriptase (TERT); Telomerase RNA template (TR); Armadillo/β-catenin-like Repeat-containing protein (ARM); Phosphoinositide 3-kinase (PI3 kinase); Mre11/Rad50/Nbs1 (MRN); End-joining (EJ); Non-homologous end-joining (NHEJ).*Arabidopsis thaliana* (At): Telomeric Repeat Binding 1-3 (AtTRB1-3); Telomere Binding Protein 1 (AtTBP1); Telomere Repeat binding Factor 1 (AtTRF1); TRF-like 2-10 (AtTRFL2-10); Suppressor of cdc thirteen homolog (AtStn1); Telomeric pathways in association with Stn1(AtTen); Conserved telomere maintenance component 1 (AtCTC1); Protection of telomeres 1a, b, c (AtPot1a, b, c); Whirly 1 (AtWhy1); Single-stranded Telomere-binding Protein 1 (AtSTEP1); Telomerase Reverse Transcriptase (AtTERT); Cajal Bodies Factor 5 (AtCBF5); RNA Recognition Motifs (AtRRM); Armadillo/β-catenin-like Repeat-containing protein (AtARM); Gene 2 (AtG2p); Metallothionein-like 2A (AtMT2A); Regulator of Telomere Elongation Helicase 1 (AtRTEL1); Exonuclease 1 (AtEXO1); Ataxia telangiectasia mutated kinase (AtARM); ATM- and RAD3-related kinases (AtATR); DNA repair protein 50 (AtRad50); Meiotic recombination 11 (AtMre11); DNA repair protein 1 (AtRad1); Excision repair cross-complementation 1 (AtERCC1); X-ray repair cross-complementing 1 (XRCC1); Nijmegen breakage syndrome 1 (AtNbs1); Poly(ADP-Ribose) polymerase 1, 2 (AtPARP1, 2). *Oriza sativa* (Os): Telomere Repeat Binding Factor 1 (OsTRBF1); Rice Telomere Binding Protein 1 (RTBP1); Anther Indehiscence 1 (OsAID1); Telomerase Reverse Transcriptase (OsTERT). *Zea mays* (Zm): Single Myb Histone (ZmSMHs); Initiator-binding protein 1 (ZmIBP1); Initiator-binding protein 2 (ZmIBP2); Terminal acidic SANT 1 (ZmTacs1); Protection of telomeres 1a, b (ZmPot1a, b); Telomerase Reverse Transcriptase (ZmTERT). *Nicotiana glutinosa/tabacum/sylvestris* (Ng/Nt/Ns): Telomere Repeat binding Factor 1 (NgTRF1); Telomeric ssDNA binding protein 1-3 (NtGTBP1-3); Telomerase Reverse Transcriptase (Ng/NtTERT). *Hordeum vulgare* (Hv): Protection of telomeres 1(HvPot1); Whirly 1 (HvWhy1). *Solanum lycopersium/tuberosum* (Le/St): Telomere Binding Protein1 (LeTBP1); Protection of telomeres 1 (StPot1). *Cestrum parqui* (Cp): Telomere Binding Protein1 (CpTBP1); Protection of telomeres 1 (CpPot1). *Petroselinum crispum* (Pc): Myb-like protein 1 (PcMYB1); Box P-binding Factor 1 (PcBPF-1).*

**Table 2:** Telomeric and putative telomeric dsDNA- and ssDNA-binding proteins from *Arabidopsis thaliana*.

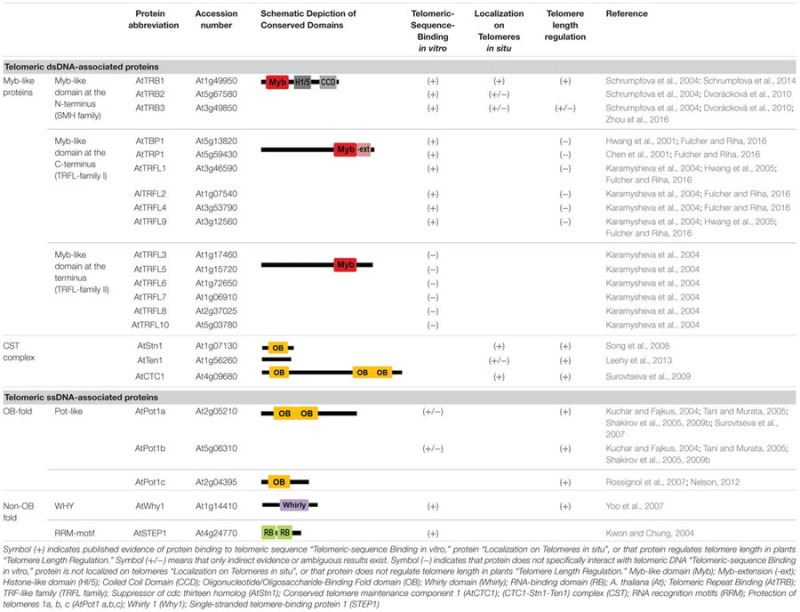

### Telomeric dsDNA-Associated Proteins

#### Myb-like Proteins

In plants, telomeric dsDNA sequence binding proteins with a Myb-like domain of a telobox (short telomeric motif) type can be classified into three main groups: (i) with a Myb-like domain at the N-terminus (SMH family), (ii) with a Myb-like domain at the C-terminus (TRFL family), and (iii) with a Myb-like domain at the C-terminus (AID family; reviewed in Peska et al., 2011; Du et al., 2013).

**FIGURE 3:**
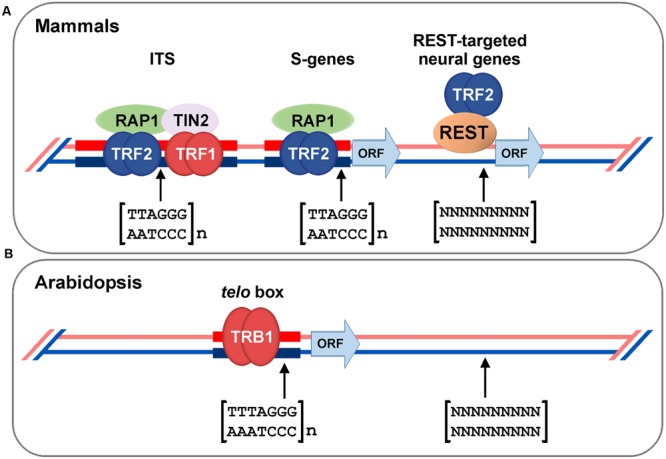
**Association of shelterin proteins with extra-telomeric sequences. (A)** Mammalian telomere-binding proteins TRF1/2, TIN2, or Rap1 associate not only with terminally localized telomeric repeats but also with interstitial telomeric sequences (ITS) and satellite repeats (Krutilina et al., 2001; Mignon-Ravix et al., 2002; Simonet et al., 2011; Yang et al., 2011). Mouse Rap1, together with TRF2, acts as a gene transcription regulator, including subtelomeric-gene (S-gene) silencing, and also binds to non-coding genomic regions enriched with TTAGGG repeats (Martinez et al., 2010). Moreover, TRF2 regulates neuronal genes by interaction of TRF2 with repressor element 1-silencing transcription factor (REST; Zhang et al., 2008). **(B)** *Arabidopsis* TRB1 protein was found not only as a component of the telomeric interactome (Schrumpfova et al., 2014), but also as a factor associated with 5′ flanking regions (mostly comprising the *telo* box) of translation machinery genes (Schrumpfova et al., 2016; Zhou et al., 2016).

The first group of proteins, with a Myb-like domain at the N-terminus, also contains a central histone-like domain with homology to the H1 globular domain found in the linker histones H1/H5, and is therefore called the SMH family (Marian et al., 2003; Schrumpfova et al., 2004). Proteins with an SMH motif are plant-specific but are well conserved throughout the plant kingdom (e.g., eudicots, monocots, moss, or red algae; Du et al., 2013). In *A. thaliana*, there are five members of the SMH family, named telomere repeat binding (AtTRB) proteins (Marian et al., 2003; Schrumpfova et al., 2004). AtTRB1 protein specifically binds plant telomeric repeats through a Myb-like domain *in vitro* (Mozgova et al., 2008), co-localizes with telomeres *in situ*, and physically interacts with AtTERT (**Figure 2B**). Moreover shortening of telomeres was observed in *attrb1* knockout mutants (Schrumpfova et al., 2014). Also other members of this family, AtTRB2 and AtTRB3 (previously named AtTBP3 and AtTBP2, respectively; Schrumpfova et al., 2004), bind telomeric dsDNA as well as telomeric ssDNA *in vitro* as homo- or heteromultimers (Schrumpfova et al., 2004; Mozgova et al., 2008; Hofr et al., 2009; Lee W.K. et al., 2012; Yun et al., 2014). In *Arabidopsis*, AtTRB1 protein physically interacts via its histone-like domain with AtPot1b (Schrumpfova et al., 2008), an *A. thaliana* homolog of the G-overhang binding protein Pot1, and a component of an alternative telomerase holoenzyme complex (Tani and Murata, 2005; He et al., 2006; Surovtseva et al., 2007). Also other members of SMH family proteins in land plants show telomeric dsDNA binding capability: e.g., *Oryza sativa* OsTRBFs (Byun et al., 2008) or *Z. mays* ZmSMHs (Marian et al., 2003). In addition, proteins with Myb-like domain of a telobox type in plants, adopt distinct non-telomeric functions, e.g., PcMYB1 from *Petroselinum crispum* acts only as a transcription factor (Feldbrugge et al., 1997). Recently it was shown that AtTRB1 from *A. thaliana* was not only telomere- and telomerase-binding but was also associated, *in vivo*, with promoters, mostly with a *telo* box motif of translation machinery genes (**Figure 3**; Schrumpfova et al., 2016). The AtTRB1 association with *telo* box motif was then proven by Zhou et al. (2016). Moreover AtTRB proteins seem to have a new role as chromatin modulators: AtTRB1 competes with LIKE HETEROCHROMATIN PROTEIN 1 (AtLHP1) to maintain downregulation of polycomb group (PcG) target genes (Zhou et al., 2016) and protein AtTRB2 directly interacts with histone deacetylases, HDT4 and HDA6, *in vitro* and *in vivo* (Lee and Cho, 2016). Deacetylase activity of HDT4 (Lee and Cho, 2016) and HDA6 (To et al., 2011) against H3K27ac, could be important for subsequent methylations of H3K27me3, that is among others target also for AtLHP1. Taken together, two lines of evidence classify the AtTRB proteins as novel epigenetic regulators that potentially impact transcription status of thousands of genes: (i) association of AtTRB1 with *telo* box DNA motif (Schrumpfova et al., 2016; Zhou et al., 2016) that is linked with PcG protein pathway (Deng et al., 2013; Wang et al., 2016; Zhou et al., 2016), (ii) involvement of AtTRB proteins in control of H3K27 epigenetic modifications (Lee and Cho, 2016; Zhou et al., 2016), that are also connected with PcG chromatin remodelers.

The second group of proteins, with a Myb-like domain at the C-terminus, is also named TRFL. However a TRFL Myb-like domain alone is not sufficient for telomere binding and requires a more extended domain—Myb-extension (Myb-ext)—for telomeric dsDNA interactions *in vitro* (Karamysheva et al., 2004; Ko et al., 2008). Consequently, two families of TRFL can be distinguished: TRFL family 1 with a Myb-ext, whose protein members bind telomeric dsDNA *in vitro*, and TRFL family 2 without a Myb-ext, whose protein members do not bind telomeric dsDNA specifically *in vitro* and they are usually not considered as telomeric proteins (Karamysheva et al., 2004). The first identification of a TRFL family protein from *O. sativa*—telomere-binding protein 1 (OsRTBP1; Yu et al., 2000) was soon followed by numerous other TRFL members: e.g., *Nicotiana glutinosa* (NgTRF1; Yang et al., 2003), *Solanum lycopersicum* (LeTBP1; Moriguchi et al., 2006), *A. thaliana* (AtTBP1, AtTRP1, AtTRFL2-10; Chen et al., 2001; Hwang et al., 2001; Karamysheva et al., 2004), *Cestrum parqui* (CpTBP; Peska et al., 2011). Even though *O. sativa* or *N. glutinosa* mutants for TRFL members exhibited markedly longer telomeres (Yang et al., 2004; Hong et al., 2007), in *A. thaliana*, a knockout of AtTRP1, member of TRFL family 1 with a Myb-ext, did not change telomere length significantly (Chen et al., 2005). Even multiple knock-out plant, deficient for all six proteins from TRFL family 1 in *A. thaliana* (AtTBP1, AtTRP1, AtTRFL1, AtTRFL2, AtTRFL4, and AtTRF9) did not exhibit changes in telomere length, or phenotypes associated with telomere dysfunction (Fulcher and Riha, 2016). Thus, although the AtTRFL proteins from *A. thaliana* specifically bind telomeric DNA *in vitro* and an interaction between AtTRP1 and AtKu70 was observed, suggesting a putative telomere function (**Figure 2B**; Kuchar and Fajkus, 2004), no functional evidence exists for their role at telomeres. Another member of this family—ZmIBP2 (initiator-binding protein) protein—binds not only telomeric repeats (Moore, 2009), but was originally identified as a promoter binding ligand (Lugert and Werr, 1994). Moreover, some members of this Myb-like family were identified exclusively based on their ability to bind promoter regions of certain genes: ZmIBP1 (Lugert and Werr, 1994), PcBPF-1 (box P-binding factor) from *P. crispum* (da Costa e Silva et al., 1993) or CrBPF from *Catharanthus roseus* (van der Fits et al., 2000).

The third group with a Myb-like domain at the C-terminus (AID family) contains only a few members. The AID family is named according to anther indehiscence 1 (AID) protein from *O. sativa*—OsAID1 (Zhu et al., 2004). OsAID1 was initially identified as being involved in anther development. Another member of this family—ZmTacs1 (terminal acidic SANT) from *Z. mays*—may function in chromatin remodeling within the meristem (Marian and Bass, 2005).

In an affinity pull-down technique, 80 proteins from *O. sativa* were identified for their ability to bind to a telomeric repeat (He et al., 2013). Among them, two of three previously reported proteins from the SMH family–OsTRBF1 and OsTRBF2 (Byun et al., 2008), and one protein with a Myb-domain at the C-terminus (AID family)—OsAID1 (Zhu et al., 2004; Du et al., 2013) were demonstrated, while no member with a Myb-domain at the C-terminus of the TRFL family could be found. From other ribonucleoproteins or RNA-binding proteins with putative telomere association, two homologs of *N. tabacum* telomeric ssDNA binding protein NtGTBP1 (Os08g0492100 and Os08g0320100), with RNA recognition motifs (RRM; see below; Lee and Kim, 2010), were also identified.

Telomere-binding proteins in budding yeast (yRap1) or in mammals (TRF1, TRF2, Rap1, and TIN2) are associated with extra-telomeric sequences and thus participate in additional roles, e.g., gene activation and repression, DNA replication, heterochromatin boundary-element formation, creation of hotspots for meiotic recombination and chromatin opening (**Figure 3A**; Morse, 2000; Smogorzewska et al., 2000; Krutilina et al., 2001; Zhang et al., 2008; Martinez et al., 2010; Simonet et al., 2011; Yang et al., 2011; Maï et al., 2014; Ye et al., 2014).

#### CST Complex

An evolutionary conserved trimeric protein complex named CST (Cdc13/CTC1–Stn1–Ten1) is, similarly to Myb-like proteins, involved in several stages of telomere end formation. In yeast, these OB-fold proteins are required for recruitment of telomerase and DNA polymerase α to the chromosomal termini, and thus coordinate G-overhang extension by telomerase with the fill-in synthesis of the complementary C-strand (Qi and Zakian, 2000; Grossi et al., 2004; Giraud-Panis et al., 2010; Wellinger and Zakian, 2012). In mammals, CST is primarily involved in the rescue of stalled replication forks at the telomere and elsewhere in the genome, and limits telomerase action at individual telomeres to approximately one binding and extension event per cell cycle (**Figure 2A**; Chen et al., 2012; Stewart et al., 2012; Chen and Lingner, 2013; Kasbek et al., 2013).

In *A. thaliana*, a mutation in any CST subunits leads to severe morphological defects and is accompanied by a decrease in telomere length, single-strand G-overhang elongation, mostly subtelomere–subtelomere chromosomal fusions and the appearance of extra-chromosomal telomeric circles. Plants lacking Suppressor of cdc thirteen homolog (AtStn1) or Conserved telomere maintenance component 1 (AtCTC1) exhibit no change in telomerase activity whereas telomerase activity was elevated in *atten1* mutants (Song et al., 2008; Surovtseva et al., 2009; Leehy et al., 2013). Although circumstantial evidence indicates that CST in plants is needed for telomere integrity, clear evidence is absent that would show any direct physical interaction of any component of the CST complex with plant telomeric DNA. As *Arabidopsis* AtCTC1 interacts with the catalytic subunit of DNA polymerase α (ICU2) *in vitro* (Price et al., 2010) and *atstn1* mutant phenotypes can be partially phenocopied by impairment of DNA polymerase α, it was recently suggested that seemingly specific function(s) of CST in telomere protection may rather represent unique requirements for efficient replication of telomeric DNA (**Figure 2B**; Derboven et al., 2014). It seems that the CST complex controls access of telomerase, end-joining recombination and the ATR-dependent (ATM and Rad3-related) DNA damage response pathway at the chromosomal ends in wild-type plants (Boltz et al., 2012; Leehy et al., 2013; Amiard et al., 2014).

### Telomeric ssDNA-Associated Proteins

#### Proteins with OB-fold

The telomeric G-rich overhang is evolutionarily conserved and is a substrate for ssDNA binding proteins. The majority of ssDNA binding proteins bind through OB motifs (OB-fold) and are required for both chromosomal end protection and regulation of telomere length, e.g., telomere-binding protein subunit alpha/beta (TEBPαβ) from *Oxytricha nova* (telomere end binding protein; Price and Cech, 1987), Cell division cycle 13 (Cdc13p) from *S. cerevisiae* (Garvik et al., 1995) and Pot1, are present in diverse organisms including human, mouse, chicken, or *S. pombe* (**Figure 2A**; Baumann and Cech, 2001; Lei et al., 2002; Wei and Price, 2004; Wu et al., 2006). In *A. thaliana*, three Pot-like proteins have been named AtPot1a, AtPot1b, AtPot1c (Kuchar and Fajkus, 2004; Rossignol et al., 2007; previously also named as AtPOT1-1, AtPOT1-2 (Tani and Murata, 2005) or AtPot1, AtPot2 (Shakirov et al., 2005; see Rotkova et al., 2009 for an overview). However, descriptions of their functions and binding properties are not unanimously agreed. While a very weak, but specific affinity of AtPot1a and AtPot1b for plant telomeric ssDNA was originally described (Shakirov et al., 2005), later these authors could not demonstrate AtPot1a and AtPot1b binding to telomeric ssDNA *in vitro* (Shakirov et al., 2009a,b). Nevertheless, stable telomeric ssDNA binding was observed for two full-length plant Pot1 proteins: OlPot1 from the green alga *Ostreococcus lucimarinus* as well as for ZmPot1b from *Z. mays* (Shakirov et al., 2009b). Although Pot1 proteins from plant species as diverse as *Hordeum vulgare* (HvPot1; barley), *Populus trichocarpa* (poplar), *Helianthus argophyllus* (sunflower), *Selaginella moellendorffii* (spikemoss), *Gossypium hirsutum* (cotton), *Pinus taeda* (pine), *Solanum tuberosum* (StPot1; potato), *Asparagus officinalis* and *Z. mays* (ZmPot1a) failed to bind telomeric DNA when expressed in a rabbit reticulocyte lysate expression system *in vitro* and subjected to an electrophoretic mobility shift assay (Shakirov et al., 2009b), binding of plant Pot1 proteins to telomeric DNA under native conditions cannot be excluded. Plants expressing AtPot1a truncated by an N-terminal OB-fold, showed progressive loss of telomeric DNA. In contrast, telomere length was unperturbed in plants expressing analogously trimmed AtPot1b, although overexpression of C-terminally truncated AtPot1b resulted in telomere shortening (Shakirov et al., 2005).

AtPot1a binds AtStn1 and AtCTC1 proteins (**Figure 2B**; Renfrew et al., 2014), associates with an N-terminally spliced variant of AtTERT (AtTERT-V(I8)) (Rossignol et al., 2007), TER1, one of the RNA subunits of *Arabidopsis* telomerase, and is required for maintenance of telomere length *in vivo* (Surovtseva et al., 2007). AtPot1b directly interacts with Myb-like proteins AtTRB1-3 from the SMH family (Schrumpfova et al., 2008), and associates with TER2 and TER2s, putative alternative RNA subunits of telomerase that negatively regulate the function of active telomerase particles (TER1-AtTERT; Cifuentes-Rojas et al., 2012). Nevertheless, AtPot1b does not seem to substantially contribute to telomere maintenance (Cifuentes-Rojas et al., 2012). Pot1-like proteins were also identified in plants with unusual telomeres (e.g., CpPot1 protein in *C. parqui*; Peska et al., 2008).

#### Non-OB-fold Telomeric ssDNA Binding Proteins

The transcriptional activator protein Whirly 1 (Why1), from a small protein family found mainly in land plants (Desveaux et al., 2000, 2002; Krause et al., 2005), was also identified in a fraction of telomere-binding proteins in *A. thaliana*, and an *atwhy1* knockout mutant appeared to have shorter telomeres (Yoo et al., 2007). While proteins from *A. thaliana* (AtWhy1; Yoo et al., 2007) and from *H. vulgare* (HvWhy1; Grabowski et al., 2008) were found to bind plant telomeric repeat sequences *in vitro*, diverse organelle localization of other Why family members from *O. sativa, A. thaliana, S. tuberosum* (Krause et al., 2005; Schwacke et al., 2007) and proposed binding to ssDNA of melted promoter regions (Desveaux et al., 2002), rather indicate a role in communication between plastid and nuclear genes encoding photosynthetic proteins (Foyer et al., 2014; Comadira et al., 2015).

A truncated derivative of chloroplast RNA-binding protein (AtCP31) with RRMs from *A. thaliana*, named AtSTEP1 (single-stranded telomere-binding protein 1), localizes exclusively to the nucleus, specifically binding single-stranded G-rich plant telomeric DNA sequences and inhibiting telomerase-mediated telomere extension (Kwon and Chung, 2004).

A protein identified by gel mobility shift assay that specifically binds the G-strand of telomeric ssDNA from *N. tabacum* (NtGTBP1) also contains a tandem pair of RRMs (Hirata et al., 2004). NtGTBP1 is not only associated with telomeric sequences, as well as two additional GTBP paralogs (NtGTBP2 and NtGTBP3), but also inhibits telomeric strand invasion *in vitro* and leaves of knockdown tobacco plants contained longer telomeres with frequent formation of extrachromosomal t-circles (Lee and Kim, 2010). These observations correspond to a previously detected protein from tobacco nuclei that binds G-rich telomeric strands and reduces accessibility to telomerase or terminal transferase (Fulneckova and Fajkus, 2000).

In addition to the above described proteins, various telomeric ssDNA binding proteins have also been reported in nuclear extracts from *Glycine max, A. thaliana, O. sativa*, or *Vigna radiata* (Zentgraf, 1995; Kim et al., 1998; Lee et al., 2000; Kwon et al., 2004). However, precise characterization of these proteins, identified by gel mobility shift assay, is mostly missing.

### DNA Repair Proteins and Telomeres

Ku in plants, as well as in other eukaryotes, is a highly conserved complex, consisting of two polypeptides (Ku70 and Ku80; Mimori et al., 1981). Due to its high affinity for DNA ends, Ku has a generally conserved role across species in protecting DNA from nucleolytic degradation. Ku is important for several cellular mechanisms: the DNA double-stranded break (DSB) repair pathway by the Ku-dependent non-homologous end-joining (NHEJ) pathway, the DNA damage response machinery, or protection of telomere ends from being recognized as DSBs, thereby preventing their recombination and degradation (reviewed in Fell and Schild-Poulter, 2015). Human Ku directly interacts not only with the shelterin proteins hTRF1, hTRF2, and hRap1, but also with telomerase subunits hTERT and hTR (RNA template; reviewed in Fell and Schild-Poulter, 2015). In contrast to a massive loss of telomeric DNA that was observed in human cells (Wang et al., 2009), mutations in Ku70 and Ku80 in the dicotyledonous *A. thaliana*, as well as in the monocotyledonous *O. sativa*, resulted in longer telomeres, suggesting their conserved role in the negative regulation of plant telomerase (Bundock et al., 2002; Riha et al., 2002; Gallego et al., 2003; Hong et al., 2010). On the other hand, severe developmental defects were observed in *O. sativa osku70* knockout mutants, but a similar mutation in *A. thaliana atku70* showed no effect on plant development (Bundock et al., 2002; Hong et al., 2010). In *S. latifolia* and *A. thaliana*, Ku contributes to the integrity of blunt-ended telomeres by protecting them from nucleolytic resection (Kazda et al., 2012). AtKu specifically interacts with AtTRP1 protein (see above; **Figure 2B**; Kuchar and Fajkus, 2004) and also assembles with TER2 and TER2_S_ into alternative telomerase complexes that cannot sustain telomere repeats on chromosomal ends (Cifuentes-Rojas et al., 2012).

The mammalian shelterin complex is involved in the repression of the primary signal transducers of DNA breakage, two phosphatidylinositol-3-kinase-like (PI3K) protein kinases: ataxia telangiectasia mutated (ATM) and ATM- and RAD3-related (ATR) kinases. Mice TRF2 acts mainly to protect telomeres against ATM activation (Celli and de Lange, 2005) and POT1 is principally involved in repression of the ATR pathway (Denchi and de Lange, 2007; Guo et al., 2007). Short telomeres in telomerase-deficient plants activate both the AtATM and AtATR, whereas absence of members of the CST complex initiates only AtATR-dependent, but not AtATM-dependent DNA damage response (Amiard et al., 2011; Boltz et al., 2012). In mammals as well as in other organisms, DSBs activate ATM kinase in a manner dependent on the meiotic recombination 11 (Mre11), DNA repair protein 50 (Rad50), and Nijmegen breakage syndrome 1 (Nbs1) named MRN complex. The MRN complex has been found to associate with telomeres and contributes to their maintenance (reviewed in Lamarche et al., 2010). *A. thaliana* AtRad50 mutant plant cells show a progressive shortening of telomeric DNA (Gallego and White, 2001), while in AtMre11 mutant plants, telomere lengthening was observed (Bundock and Hooykaas, 2002). Contrary to these observations, the absence of the third MRN subunit, AtNbs1, does not affect the length of telomeres (Najdekrova and Siroky, 2012).

*A. thaliana* plants mutated in XPF (xeroderma pigmentosum group F-complementing) and ERCC1 (excision repair cross-complementation group 1) orthologs that form a structure-specific endonuclease essential for nucleotide excision repair (known as AtRad1 and AtERCC1), develop normally and show wild-type telomere length. However, in the absence of telomerase, mutations in either of these genes induce a significantly earlier onset of chromosomal instability, thus indicating a protective role of AtERCC1/AtRad1 against a 3′ G-strand overhang invasion of interstitial telomeric repeats (Vannier et al., 2009). In addition to the Ku proteins that are involved in Ku-dependent NHEJ, an alternative Ku-independent NHEJ pathway was described (reviewed in Decottignies, 2013). Members of the poly(ADP-ribose) polymerase family play a role not only in the base excision repair pathway and the backup-NHEJ KU-independent pathway (Decottignies, 2013) but were also studied in the context of telomere maintenance, association with shelterin proteins or modulation of telomerase activity (Smith et al., 1998; Cook et al., 2002; Beneke et al., 2008). However, analysis of *Arabidopsis* orthologs AtPARP1/AtPARP2 (poly(ADP-Ribose) polymerase) has revealed that, unlike in humans, AtPARPs play a minor role in telomere biology (Boltz et al., 2014). It was proposed that DSB repair pathways in *A. thaliana* are hierarchically organized and the Ku-dependent NHEJ restricts access and action of other DSB repair processes (Charbonnel et al., 2010, 2011). Furthermore the end-joining recombination proteins (AtKU80, AtXRCC1, AtRad1) restrict telomerase activity at deprotected telomeres (Amiard et al., 2014). It was found recently that structure-specific endonucleases AtMUS81 (MMS and UV-sensitive protein 81) and AtSEND1 (single-strand DNA endonuclease 1), which presumably act to repair potentially toxic structures produced by DNA replication and recombination, are essential for telomere stability in *Arabidopsis*. Combined absence of these endonucleases results in increased occurrence of histone γ-H2AX foci in S-phase and in loss of telomeric DNA (Olivier et al., 2016).

## Plant Telomerase

Telomere length in plants and various other organisms is maintained by telomerase, a specialized reverse transcriptase which, in addition to its catalytic subunit (TERT), carries its own RNA template (TR) and elongates telomeric tracts at the chromosomal terminus (Blackburn and Gall, 1978; Fajkus et al., 1996).

TERT subunits consist of an N-terminal portion with telomerase-specific motifs important for binding the telomerase RNA subunit, catalytic domains with the telomerase reverse transcriptase (RT) motifs essential for enzyme activity, and the C-terminal extension, which is highly conserved among plants as well as vertebrates (Sykorova and Fajkus, 2009). Although most eukaryotes harbor only a single TERT gene, in the allotetraploid *N. tabacum* there are three NtTERT gene variants inherited from its diploid progenitor species *N. sylvestris* and *Nicotiana tomentosiformis.* All three NtTERT gene variants are transcribed.

Alternative splicing provides a major source of protein diversity within a given organism. Alternatively spliced variants of TERT transcripts with out-of-frame and/or in-frame mutations were identified not only in humans, mouse, chicken, or *Xenopus* (reviewed in Hrdlickova et al., 2006), but also in many plant species, e.g., *A. thaliana, Z. mays* (ZmTERT), *O. sativa* (OsTERT), *Iris tectorum*, and tobacco [with human-type (TTAGGG) telomere motif; reviewed in Sykorova and Fajkus, 2009; Sykorova et al., 2012]. Isoforms generated by alternative splicing may show changes or loss of specific function(s) or subcellular localization of the respective product, or could be functionally important, as was suggested for the *A. thaliana* variant AtTERT V(I8) that exclusively interacts with AtPot1a (Rossignol et al., 2007).

It has been proposed that human telomerase is subjected to posttranslational regulation such as phosphorylation (Kang et al., 1999). Putative phosphorylation sites were detected in the OsTERT sequences from *O. sativa* (Oguchi et al., 2004) or *N. tabacum* BY-2 cells (Yang et al., 2002) but not in AtTERT from *A. thaliana* (Oguchi et al., 2004).

### Telomerase-Associated Proteins

Rich protein interactomes of yeast, mammalian or *Ciliate* TERT have been described, including the Ku heterodimer (Chai et al., 2002), HSP90 (heat-shock protein of 90 kDa; Holt et al., 1999; Grandin and Charbonneau, 2001), ATPases pontin and reptin (Venteicher et al., 2008), TEP1 (telomere protein 1; Harrington et al., 1997), and many others, in a broad study (Fu and Collins, 2007) and reviewed in a constantly updated telomerase database (Podlevsky et al., 2008).

In AtTERT, a mitochondrial targeting signal, multiple nuclear localization signals or a nuclear export signal have been reported (Zachova et al., 2013). As AtTERT protein and its domains localize mainly within the nucleus and the nucleolus (Zachova et al., 2013), it can be assumed that most interacting protein partners relevant to telomeric functions will be found among nuclear or nucleolar proteins.

In plants, a limited number of proteins that directly interact with TERT have been described. It was demonstrated by various direct methods that AtTRB proteins, a group of plant homologs of human TRF proteins with a Myb-domain at the N-terminus (see above), physically interact with N-terminal domains of AtTERT (**Figure 2B**; Schrumpfova et al., 2014). A mediated interaction between AtTRP1 protein that belongs to the TRFL family, and AtTERT, was also observed (Schrumpfova et al., 2014). Moreover, the N-terminal part of AtTERT exclusively interacts with AtPot1a but not AtPot1b (Rossignol et al., 2007). Also various proteins with an RRM-motif (AtRRM), an ARM-motif (armadillo/β-catenin-like repeat-containing protein; AtARM), metallothionein-like (AtMT2A), or RNA-binding (AtG2p) motifs were found as AtTERT interacting partners in *A. thaliana* (Lee L.Y. et al., 2012; Dokládal et al., 2015).

Indirect regulation of TERT by various proteins or hormones was further described in plants. In tobacco cell culture, phytohormones such as auxin or abscisic acid regulate phosphorylation of telomerase protein, which is required for the generation of a functional telomerase complex (Tamura et al., 1999; Yang et al., 2002). In *A. thaliana*, reduced endogenous concentrations of auxin in telomerase activator 1 (AtTAC1) mutant plants blocks the ability of this zinc-finger protein to induce AtTERT. However, AtTAC1 does not directly bind the AtTERT promoter (Ren et al., 2004, 2007). A minimal promoter region for AtTERT was proposed using a set of T-insertion mutant lines in the protein-coding region of the *AtTERT* gene or in lines with insertions at the 5′ end of *AtTERT* (Fojtova et al., 2011). Moreover T-DNA insertions in the region upstream of the ATG start of *AtTERT* also led to the activation of putative regulatory elements (Fojtova et al., 2011).

In vertebrates, only one TR per organism was described. The folding of the TR molecule offers interaction sites for various associating cofactors such as dyskerin, Ku, nucleolar protein 10 (NOP10), H/ACA ribonucleoprotein complex subunit 1 (GAR1), or subunit 2 (NHP2; Ting et al., 2005; for review, see Kiss et al., 2010). A single TR was also described among Brassicaceae family plants. However, in *A. thaliana*, two TRs were detected—TER1 and TER2, and the latter may be alternately spliced to a TER2s form (Beilstein et al., 2012). The *Arabidopsis* homolog of human dyskerin, named AtCBF5 (alias AtNAP57), is located within nucleoli and Cajal bodies (Lermontova et al., 2007), associates with active telomerase, and weakly with AtPOT1a, but not AtTERT or AtKu70 (Kannan et al., 2008).

### Telomerase-Independent Processes in Plant Telomere Dynamics

Compared to the human model, knowledge of individual protein contributions to the maintenance of telomere length/ accessibility/folding in plants or telomerase biogenesis/regulation is still very limited. The process of telomere maintenance is complicated by the fact that besides the widespread system of telomere maintenance by telomerase (Fajkus et al., 1996; Heller et al., 1996) in plants as well as in other organisms, in the absence of telomerase, telomeres can be elongated by recombination-dependent and telomerase-independent alternative telomere lengthening (ALT) mechanisms (Fajkus et al., 2005). Moreover, in plants, the ALT events appear to participate in early plant development (Ruckova et al., 2008). It was shown that AtKu70 deficiency facilitates engagement of ALT lengthening in *A. thaliana* (Zellinger et al., 2007) and that ALT was suppressed in the absence of ATM protein (Vespa et al., 2007).

Telomeric DNA of higher eukaryotes, including plants, is associated not only with specific proteins, but also with histone complexes that form nucleosomes (**Figure 2**; reviewed in Dvořáčková et al., 2015). In various organisms, as well as in plants, telomeric nucleosomes display an unusually short periodicity (157 bp in length), usually 20–40 bp shorter than bulk nucleosomes of the corresponding organism (Fajkus et al., 1995; Fajkus and Trifonov, 2001; reviewed in Pisano et al., 2008). Moreover, the plant telomeric repeat (CCCTAAA) is a natural target for plant-specific asymmetric methylation (Cokus et al., 2008) that was shown to be mediated by an siRNA pathway (Vrbsky et al., 2010). Analysis of telomeres in *A. thaliana* (Vrbsky et al., 2010) and *N. tabacum* (Majerova et al., 2011) has demonstrated that telomeric histones were associated with both heterochromatin- and euchromatin-specific marks. Recent data strongly support the involvement of various epigenetic mechanisms (DNA methylation, posttranslational modifications of histones, nucleosome assembly or levels of telomere-repeat containing RNA) in maintenance of telomere stability (reviewed in Dvořáčková et al., 2015) thus demonstrating complexity of telomere regulation.

## Conclusion

The need for protection of chromosomal termini remains conserved across most species. Nevertheless, an extraordinary plasticity of mechanisms protecting telomeres has been described among various organisms (reviewed in Giraud-Panis et al., 2013). While individual capping proteins can differ greatly, common features such as homologous binding domains, structures, or interacting partners exist between seemingly different capping systems. Plant systems show certain distinct features of telomere maintenance, including the reversible regulation of telomerase in somatic cells and the absence of developmental telomere shortening (Fajkus et al., 1998; Riha et al., 1998). These distinctions promote further efforts to elucidate plant telomere interactomes. Only recently the first complexes of telomere-binding proteins were demonstrated and meanwhile it seems that the plant telomere-maintenance system shares similarities with that described in mammals. For example, in *A. thaliana*, one of the most studied plant model systems: (i) the core plant telomeric dsDNA binding proteins (AtTRBs, AtTRP, etc.) contain similar Myb-domains which are also present in human TRF1 or TRF2 proteins; (ii) homologs of human telomeric ssDNA binding hPot1 (AtPOT1a-c) were described; (iii) cross-species conserved CST complexes (AtCTC1/AtTen1/AtStn1) retain its function in plants. The similarities between plant and mammalian telomeric DNA-associated proteins apply also to their roles in regulation of gene expression, which are independent of their roles in telomere capping (Lee and Cho, 2016; Schrumpfova et al., 2016; Zhou et al., 2016), as was previously described in their mammalian counterparts (reviewed in Maï et al., 2014; Ye et al., 2014). Elucidation of the composition of the plant version of shelterin and molecular dissection of its components and their roles will be important in the near future to assess the conservation and mechanisms of end-protection and end-replication processes in yeasts, plants and animals.

## Author Contributions

PPS contributed substantially to the writing of the manuscript, tables and drawing the figures; ŠS participated in preparation of tables; JF edited the manuscript. All authors read and approved the manuscript for publication.

## Conflict of Interest Statement

The authors declare that the research was conducted in the absence of any commercial or financial relationships that could be construed as a potential conflict of interest.

The handling Editor declared a current collaboration as co-Topic Editor in a Frontiers Research Topic with one of the authors, JF, and states that the process nevertheless met the standards of a fair and objective review. This was also confirmed by the Specialty Chief Editor of section Plant Cell Biology, Simon Gilroy.
